# Diagnostic Value of Neutrophil CD64 in Differentiating Scrub Typhus From Febrile Diseases

**DOI:** 10.1002/iid3.70396

**Published:** 2026-03-23

**Authors:** Jing Su, Junyi Chen, Xuan Shen, Yaoyun Zhang, Wenya Li, Jijin Qi, Qing Zhong, Tianyu Sun, Siqi Shi, Fang Zhu, Youde Yan

**Affiliations:** ^1^ Jiangsu Province (Suqian) Hospital Suqian Jiangsu China; ^2^ The Suqian Clinical College of Xuzhou Medical University Suqian Jiangsu China

**Keywords:** CD64, diagnosis, fever‐related diseases, ROC, scrub typhus

## Abstract

**Background:**

Scrub typhus, lacks specific early symptoms, and traditional serological tests have low sensitivity. This study shows that CD64 has superior diagnostic performance compared to CRP and PCT in bacterial infections.

**Methods:**

This retrospective study analyzed 242 febrile patients admitted to the Jiangsu Province (Suqian) Hospital (January 2023–December 2024), categorized into non‐specific fever (*n* = 186) and scrub typhus (*n* = 56). The CD64 expression was quantified via flow cytometry (BD FACS Canto II) within 48 h of admission. ROC analysis evaluated diagnostic performance using AUC, sensitivity, specificity, and Youden index. Mann–Whitney *U* and Chi‐square tests compared groups (SPSS v26.0), with significance at *p* < 0.05.

**Results:**

The scrub typhus group had significantly higher CD64 levels (median: 2.90 [IQR: 1.00–5.20]) than non‐specific fever (0.23 [0.10–0.58], *p* < 0.001). ROC analysis demonstrated CD64's high discriminatory power: AUC = 0.938 (95% CI: 0.882–0.995) for scrub typhus vs. non‐specific fever (sensitivity = 91.7%, specificity = 89.8%). Traditional markers like WBC and lymphocyte percentage showed high discrimination against non‐specific fever (AUC = 0.973–0.989).

**Conclusion:**

This study shows that CD64 is an efficient biomarker for differentiating scrub typhus from febrile diseases, offering crucial evidence for establishing rapid diagnostic pathways and holding value for early triage of febrile patients.

## 1 Introduction

Fever‐related diseases, a common clinical syndrome in infectious diseases, pose significant challenges for early precise diagnosis due to their complex etiology and overlapping clinical manifestations. Taking scrub typhus as an example, this disease is a tick‐borne febrile disease caused by an infection with *Orientia tsutsugamushi* [1]. The classic triad of rickettsiosis symptoms is a high fever, skin rash, and eschar. Moreover, these patients exhibit thrombocytopenia, elevated transaminases, and inflammatory markers. Tetracyclines are effective antibiotics against most rickettsioses [2].

In early stages, scrub typhus lacks specific symptoms (such as eschars), and traditional serological tests like the Weil–Felix test show low sensitivity (< 30%) within the first 7 days of illness [3]. While molecular detection methods (e.g., PCR) can improve early diagnostic rates, their high cost and reliance on specialized laboratory equipment limit their application in primary healthcare settings [4]. Diagnostic delays can lead to multi‐organ dysfunction and increased mortality risk (6%–15%) [5]. Early diagnosis and prompt treatment of scrub typhus are crucial to improve the prognosis of such patients. Therefore, identifying sensitive, rapid, and cost‐effective biomarkers to optimize the differential diagnosis of fever‐related diseases has become an urgent global public health challenge [6].

In recent years, CD64, a surface molecule on neutrophils, has gained attention as an immunological marker of infection. Upon pathogen invasion, pro‐inflammatory cytokines (e.g., IFN‐γ and TNF‐α) induce a rapid upregulation of CD64 within 6 h, with its expression intensity positively correlated with disease severity [7]. Flow cytometric quantification of CD64 has demonstrated superior diagnostic performance for bacterial sepsis compared to C‐reactive protein (CRP) and procalcitonin (PCT) [8, 9]. However, most studies have focused on single types of infections (e.g., bacterial or viral), and the diagnostic potential of CD64 for special pathogens like scrub typhus remains unexplored [10]. Recent research suggests that rickettsiae may evade immune surveillance by inhibiting host TLR signaling pathways, potentially leading to unique CD64 expression patterns [11]. Additionally, patients with chronic liver disease exhibit reduced baseline CD64 levels due to impaired Kupffer cell function, while non‐infectious fevers (e.g., autoimmune diseases) may indirectly regulate CD64 expression through the IL‐6 pathway [12, 13, 14]. These mechanisms indicate that CD64 may play a stratified diagnostic role in febrile patients with different etiologies.

This study investigates the application of CD64 in the differential diagnosis of two categories of febrile diseases: non‐specific fever and scrub typhus. The aim is to explore whether CD64 can overcome the limitations of traditional biomarkers, enabling early and highly specific diagnosis of scrub typhus, as well as to determine its cutoff values and clinical feasibility in differentiating various etiologies. The findings will provide new strategies for precise triage in patients with undifferentiated fever and may reshape biomarker‐based diagnostic pathways for infectious diseases.

## 2 Methods

### 2.1 Study Design

**FIGURE 1:**
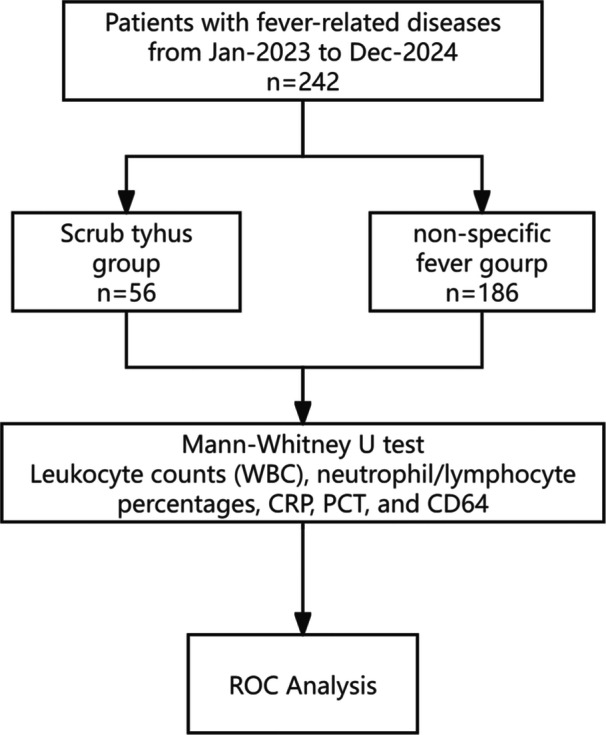
Flowchart of the study.

This retrospective study analyzed clinical data from 242 patients with fever‐related diseases admitted to the Department of Infectious Diseases at the Jiangsu Province (Suqian) Hospital (Ethics No.2025‐SR‐0154) between January 2023 and December 2024. Patients were categorized into two groups based on their diagnoses: non‐specific fever group (*n* = 186) and scrub typhus group (*n* = 56) (Figure 1).

### 2.2 Inclusion and Exclusion Criteria

Inclusion criteria: (1) Admitted to the Department of Infectious Diseases, Jiangsu Province (Suqian) Hospital between December 2023 and January 2024, with a diagnosis of a febrile disease (peak fever ≥ 38°C) and meeting any of the following diagnostic criteria: Fever group: Non‐specific fever after ruling out infectious causes (e.g., drug fever and autoimmune diseases); Scrub typhus group: Positive Weil–Felix test (OXK ≥ 1:160) or confirmed by targeted PCR. (2) Completion of CD64 flow cytometry and blood routine tests within 48 h of admission. (3) Age ≥ 18 years (to align with the target population, as the median age in the original study was 53–56 years).

Exclusion criteria: (1) Absence of complete key indicators (e.g., CD64 values, baseline blood routine tests, and diagnostic evidence); (2) Concurrent hematological malignancies or advanced solid tumors; (3) Use of immunosuppressants, cytotoxic agents, or glucocorticoids within 2 weeks before admission; (4) Hospital stay < 48 h (insufficient data for complete diagnosis and treatment); and (5) Discontinuation of treatment, self‐discharge, or transfer to another healthcare facility.

### 2.3 Data Collection

Clinical information of patients with fever‐related diseases was collected from the medical record management system and entered and managed using Epidata V.3.1 software. Baseline demographics, including age, gender, comorbidities, and medication intake were recorded. Peripheral venous blood samples were collected from all participants within 48 h of admission. CD64 expression on neutrophils was quantified by flow cytometry using a BD FACS Canto II system. Briefly, whole blood samples were stained with anti‐human CD64‐PE antibody (clone 10.1, BD Biosciences) and erythrocytes were subsequently lysed using BD FACS Lysing Solution. Neutrophils were identified based on forward and side scatter (FSC/SSC) properties, and the mean fluorescence intensity (MFI) of CD64 within this gated population was analyzed. An isotype control (Mouse IgG1‐PE) was included in each experiment to define nonspecific binding and ensure measurement specificity. All samples were processed within 2 h of collection to ensure stability. This process followed standardized protocols to ensure the reliability and consistency of the data collected.

### 2.4 Statistical Analysis

All statistical analyses were performed using SPSS version 26.0 (IBM Corp., USA), with the significance level set at *α* = 0.05. Continuous variables, confirmed to be non‐normally distributed by Shapiro–Wilk test, are expressed as median (interquartile range) [M (P25, P75)]. Comparisons between two groups were conducted using the Mann–Whitney *U* test, as our study involves two independent, non‐paired groups and the continuous variables significantly deviated from normal distribution. Categorical variables are presented as frequencies (percentages) and compared using Fisher's exact test or Chi‐square test, as appropriate. Receiver operating characteristic (ROC) curve analysis was conducted to evaluate the diagnostic performance of biomarkers. The area under the ROC curve (AUC) with its exact binomial 95% confidence interval (CI) was calculated, and its statistical significance against the null hypothesis (AUC = 0.5) was determined using the Z test. The optimal cutoff value was derived by maximizing the Youden index (sensitivity + specificity − 1).

## 3 Results

### 3.1 General Information of the Enrolled Patients

A total of 242 patients were included in the study to assess immune cell ratio differences among the groups: non‐specific fever and scrub typhus. The cohort comprised 56 patients with scrub typhus (23.1%) and 186 patients with non‐specific fever (76.9%) (Table 1). The mean ages were 57.4 ± 17.0 years for the scrub typhus group and 49.8 ± 19.8 years for the non‐specific fever group. No statistically significant differences in age and sex were observed among the groups (*p* > 0.05) (Table 1).

**TABLE 1:** Demographics and CD64 index among scrub typhus and non‐specific fever groups.

Variable	Scrub typhus (*n* = 56)	Non‐specific fever (*n* = 186)	*P*
Age, years	57.4 ± 17.0	49.8 ± 19.8	0.215
Gender, *n* (%)			0.601
Male	34 (60.7%)	106 (57.0%)	
Female	22 (39.3%)	80 (43.0%)	
**Laboratory test**			
WBC (x10^9^/L)	7.16 (4.66, 9.87)	23.9 (18.0, 31.9)	< 0.001
Neutrophil (%)	40.5 (30.1, 58.7)	81.8 (68.0, 86.8)	< 0.001
Lymphocyte (%)	51.1 (35.0, 60.8)	10.2 (7.1, 15.0)	< 0.001
CRP (ng/mL)	36.4 (27.4, 56.9)	49.7 (21.5, 107.0)	0.377
PCT (ng/mL)	0.42 (0.26, 0.78)	1.05 (0.21, 3.48)	0.098
**Indicators for diagnosing infectious diseases**			
CD64 index	2.90 (1.00, 5.20)	0.23 (0.10, 0.58)	< 0.001

### 3.2 Comparison of the Laboratory Test Between Scrub Typhus Group and Non‐Specific Fever Group

Significant differences in laboratory parameters were observed among the groups. The scrub typhus group demonstrated lower leukocyte counts (WBC:7.16 [IQR:4.66–9.87] × 10⁹/L) compared to the non‐specific fever group (23.9 [18.0–31.9] × 10⁹/L, *P*1 < 0.001) (Table 1). Neutrophil percentages were significantly reduced in scrub typhus (40.5% [30.1%–58.7%]) vs. non‐specific fever (81.8% [68.0%–86.8%], *P*1 < 0.001) (Table 1). Conversely, lymphocyte percentages were markedly elevated in scrub typhus (51.1% [35.0%–60.8%]) compared to non‐specific fever (10.2% [7.1%–15.0%], *P*1 < 0.001), but no differences were observed in CRP and PCT levels (all *p* > 0.05) (Table 1).

### 3.3 Comparison of the CD64 Index Between Scrub Typhus Group and Non‐Specific Fever Group

Significant differences in immune cell ratios were identified between the two groups. The scrub typhus group exhibited markedly higher CD64 levels compared to the non‐specific fever group (2.90 vs. 0.23, *P*1 < 0.001). No significant differences in CD64 levels were observed between the non‐specific fever group. Additionally, the scrub typhus group had the highest median CD64 value of 2.90 compared to the non‐specific fever group (Table 1).

### 3.4 Efficacy Evaluation of Laboratory Test

**FIGURE 2:**
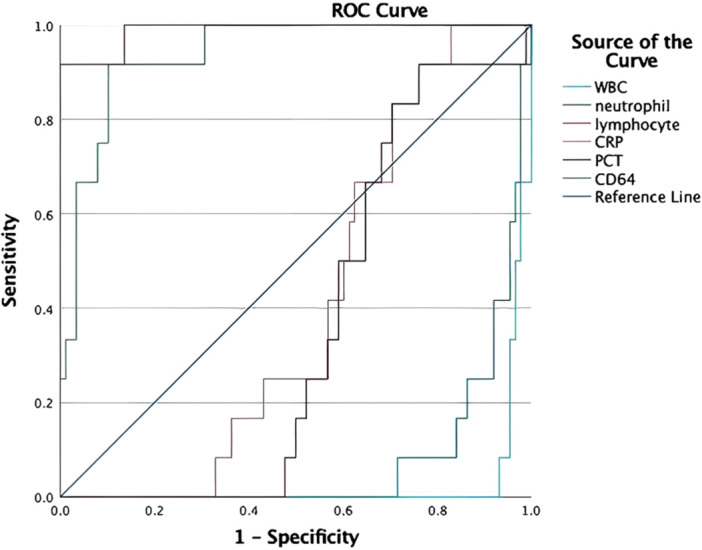
The ROC curves of classification efficacy between non‐specific fever and scrub typhus.

WBC count, neutrophil percentage and lymphocyte percentage showed differential diagnostic value in certain comparisons. In the scrub typhus vs. non‐specific fever group, WBC (AUC = 0.973, *p* < 0.001) and lymphocyte percentage (AUC = 0.989, *p* < 0.001) showed high discrimination. When WBC was 13.03 × 10⁹/L, sensitivity was 93.2% and specificity 100% (Youden index 0.932) (Table 2). When the lymphocyte percentage was 24.92%, sensitivity was 91.7%, and specificity 100% (Youden index 0.917). The neutrophil percentage also had significant discrimination (AUC = 0.922), with a cut‐off value of 63.5% (sensitivity 84.1% and specificity 91.7%) (Table 2). However, CRP and PCT showed no significant difference in the two groups (AUC = 0.592, 0.642 all *p* > 0.05) (Figure 2, Table 2).

**TABLE 2:** Detection efficacy of indexes for diagnosing scrub typhus.

Test result variable(s)	Area	*p*	95% confidence interval		Cut off	Sensitivity	Specificity	Youden
			Lower bound	Upper bound				
WBC (x10^9^/L)	0.973	0.000	0.945	1.000	13.03	0.932	1.000	0.932
Neutrophil (%)	0.922	0.000	0.863	0.981	63.50	0.841	0.917	0.758
Lymphocyte (%)	0.989	0.000	0.966	1.000	24.92	0.917	1.000	0.917
CRP (ng/mL)	0.592	0.304	0.473	0.711	82.30	0.330	1.000	0.330
PCT (ng/mL)	0.640	0.116	0.527	0.754	1.10	0.477	1.000	0.478
CD64	0.938	0.000	0.882	0.995	0.96	0.917	0.898	0.814

### 3.5 Efficacy Evaluation of CD64 Index

ROC curve analysis indicates that the CD64 index has significant diagnostic value in distinguishing scrub typhus from non‐specific fever group. In differentiating scrub typhus from non‐specific fever, CD64 shows an AUC of 0.938 (95% CI: 0.882–0.995, *p* < 0.001), with a cut‐off value of 0.96, achieving 91.7% sensitivity, 89.8% specificity, and a Youden index of 0.814. CD64 is the most reliable biomarker for differentiating scrub typhus from non‐specific fever group (Figure 2, Table 2).

## 4 Discussion

Scrub typhus (ST) is *Orientia tsutsugamushi* (OT) illness. The main source of infection is rats, and the transmission vector is trombiculid mites [15]. Larval stage mites (chiggers) cause skin lesions by biting human tissue and eventually form clinically characteristic ulcers or eschar [16]. Pathogens can be distributed to different organs or tissues of the body along blood and lymphatic circulation [17]. They infiltrate endothelial cells of different organs, such as heart, lungs, brain, liver, and kidneys from the lymph nodes [18]. Vascular endothelial damage and excessive inflammatory responses may lead to systemic poisoning symptoms, damage lesions of corresponding organs and tissues, and even multiple organ dysfunction syndrome (MODS) or death [19].

This study systematically demonstrates the diagnostic value of CD64 in differentiating scrub typhus from non‐specific fever. The scrub typhus group exhibited significantly higher CD64 levels (median: 2.90 [IQR: 1.00–5.20]) compared to non‐specific fever (0.23 [0.10–0.58], *p* < 0.001). ROC analysis confirmed its robust diagnostic efficacy: CD64 achieved an AUC of 0.938 (95% CI: 0.882–0.995) for scrub typhus vs. non‐specific fever. These findings align with CD64's role as a neutrophil activation marker in rickettsial infections, where *Orientia tsutsugamushi* triggers FcγRI (CD64)‐mediated phagocytosis and cytokine release [20, 21, 22].

Traditional markers like WBC and lymphocyte percentage also showed high discrimination for scrub typhus vs. non‐specific fever (AUC = 0.973–0.989). However, PCT and CRP showed no diagnostic value in distinguishing scrub typhus from pulmonary infection. In contrast, CD64 maintained superior performance across comparisons, supporting its specificity for scrub typhus over non‐specific fever. These complements report on CD64's reliability in sepsis diagnosis [23, 24, 25], though our cutoff values (0.93–0.96) differ from thresholds for bacterial infections, likely reflecting pathogen‐specific immune responses.

This study has several limitations. First, it employed a single‐center design with a modest sample size, which may restrict the generalizability of the findings to broader populations. Second, the different stages of infection or underlying causes in febrile patients are not adequately differentiated. Future studies can use a multicenter prospective design, combined with serial etiological detection and dynamic monitoring of clinical parameters, to more comprehensively evaluate the evolutionary characteristics and prognostic influencing factors of febrile illness.

Clinically, CD64 could reduce misdiagnosis of scrub typhus, which mimics non‐specific fever. Its high negative predictive value may guide targeted therapy, minimizing empirical antibiotic misuse. This is in line with previous studies showing CD64 outperforms traditional markers such as CRP and PCT in a range of infectious conditions [24, 26]. Moreover, studies have demonstrated CD64's stability in elderly and neonatal populations, highlighting its broader clinical applicability [8, 27].

## 5 Conclusion

This study shows that CD64 is an efficient biomarker for differentiating scrub typhus from non‐specific fever, offering crucial evidence for establishing rapid diagnostic pathways and holding value for early triage of febrile patients.

## Author Contributions

**Jing Su:** conceptualization, methodology, investigation, supervision, formal analysis, data curation, interpretation of data, writing – original draft, writing – review and editing. **Junyi Chen:** conceptualization, methodology, investigation, supervision, formal analysis, data curation, interpretation of data, writing – original draft, writing – review and editing. **Xuan Shen:** supervision, data curation, formal analysis. **Yaoyun Zhang:** supervision, data curation, formal analysis. **Wenya Li:** formal analysis. **Jijin Qi:** formal analysis. **Qing Zhong:** interpretation of data. **Tianyu Sun:** interpretation of data. **Siqi Shi:** interpretation of data. **Fang Zhu:** writing – review and editing. **Youde Yan:** writing – review and editing. All authors have read and approved the final manuscript.

## Ethics Statement

The study was approved by the Medical Ethics Committee of Jiangsu Province (Suqian) Hospital (2025‐SR‐0154) and conducted in accordance with the Declaration of Helsinki.

## Consent

All patients provided informed consent by signing an informed consent form.

## Conflicts of Interest

The authors declare no conflicts of interest.

## Policy on Using ChatGPT and Similar AI Tools

The authors did not use generative artificial intelligence in the preparation of this work.

## Supporting information

STROBE‐checklist‐v4‐combined‐PlosMedicine.

## Data Availability

The data that support the findings of this study are available from the corresponding author upon reasonable request.
